# Routes of administration for adeno-associated viruses carrying gene therapies for brain diseases

**DOI:** 10.3389/fnmol.2022.988914

**Published:** 2022-10-26

**Authors:** Kai Zhou, Jinming Han, Yafeng Wang, Yaodong Zhang, Changlian Zhu

**Affiliations:** ^1^Henan Neurodevelopment Engineering Research Center for Children, Zhengzhou Key Laboratory of Pediatric Neurobehavior, Children’s Hospital Affiliated to Zhengzhou University, Zhengzhou, China; ^2^Department of Neurology, Xuanwu Hospital, Capital Medical University, Beijing, China; ^3^Department of Hematology and Oncology, Children’s Hospital Affiliated to Zhengzhou University, Henan Children’s Hospital, Zhengzhou Children’s Hospital, Zhengzhou, China; ^4^Henan Key Laboratory of Child Brain Injury and Henan Pediatric Clinical Research Center, The Third Affiliated Hospital and Institute of Neuroscience, Zhengzhou University, Zhengzhou, China; ^5^Centre for Brain Repair and Rehabilitation, Institute of Neuroscience and Physiology, Sahlgrenska Academy, University of Gothenburg, Gothenburg, Sweden

**Keywords:** gene therapy, AAVs, serotypes, administration routes, intravenous administration, intraparenchymal injection, cerebrospinal fluid

## Abstract

Gene therapy is a powerful tool to treat various central nervous system (CNS) diseases ranging from monogenetic diseases to neurodegenerative disorders. Adeno-associated viruses (AAVs) have been widely used as the delivery vehicles for CNS gene therapies due to their safety, CNS tropism, and long-term therapeutic effect. However, several factors, including their ability to cross the blood–brain barrier, the efficiency of transduction, their immunotoxicity, loading capacity, the choice of serotype, and peripheral off-target effects should be carefully considered when designing an optimal AAV delivery strategy for a specific disease. In addition, distinct routes of administration may affect the efficiency and safety of AAV-delivered gene therapies. In this review, we summarize different administration routes of gene therapies delivered by AAVs to the brain in mice and rats. Updated knowledge regarding AAV-delivered gene therapies may facilitate the selection from various administration routes for specific disease models in future research.

## Introduction

Gene therapies exert their therapeutic effects by either removing pathologic genes or adding therapeutic genes, and such therapies can be used for various central nervous system (CNS) diseases including both genetic and neurodegenerative diseases (Hocquemiller et al., 2016). Gene therapies are usually partly or entirely composed of genetic materials (DNA or RNA) that are easily degraded in the body. Therefore, gene therapies require vehicles to deliver the genetic materials to the targeted cells or tissues. Among all used delivery vehicles, recombinant adeno-associated viruses (rAAVs) are the leading vehicle for delivering gene therapies into the CNS due to their safety, CNS tropism, and long-term therapeutic effect (Hastie and Samulski, 2015; Hudry and Vandenberghe, 2019).

Adeno-associated viruses (AAVs) are non-pathogenic viruses comprising an icosahedral capsid of ∼26 nm in diameter and a single-strand DNA of ∼4.7 kb, and they can be found in many mammals, including humans and non-human primates (Muzyczka and Berns, 2001). rAAVs are recombinant AAVs with exogenous DNA of interest. AAVs were first discovered in the 1960s (Blacklow et al., 1967, 1968), and since then rAAVs have been proposed and extensively tested as gene delivery vehicles in preclinical and clinical trials. rAAVs can be used to deliver therapeutic genes for correcting monogenetic diseases, silencing a mutated toxic gene, and releasing neurotrophic factors for multifactorial CNS diseases (Piguet et al., 2017). However, rAAVs cannot proliferate and replicate and thus they can only be expressed in the initially transduced cells, and thus repeated rAAV drug deliveries are sometimes needed (Samulski et al., 1987; Xiao et al., 1997). To date, two rAAV drugs have been approved by the FDA, namely Luxturna for inherited retinal dystrophy and Zolegensma for spinal muscular atrophy. In addition, rAAV-delivered gene therapies have been tested in preclinical experiments and in clinical trials for other tissues, including the liver, muscle, blood, and brain (Keeler and Flotte, 2019).

Different serotypes, such as AAV1, AAV2, AAV5, AAV8, AAV9, and AAV rhesus isolate10 (AAVrh.10), have shown efficacy in transducing neurons, while they have limited efficacy in transducing other glia cells (Burger et al., 2004; Cearley and Wolfe, 2006; Dodiya et al., 2010). In addition, different strategies have been used to identify or generate new AAVs with brain and other tissue tropisms, including natural discovery, capsid design, directed evolution, and *in silico* reconstruction (Keeler and Flotte, 2019; Wang et al., 2019). Many extracellular and intracellular trafficking processes are critical for successful gene delivery, such as cell-specific targeting and endosome escape, and these have been detailed and summarized in other reviews (Wang et al., 2019). Moreover, the tropism and transduction rates of different rAAV serotypes also depend on the tested species, brain regions, and administration routes (Pillay and Carette, 2017). The administration routes of rAAV delivery for clinical trials and large animals such as cats, dogs, and non-human primates have been examined in other reviews (Hudry and Vandenberghe, 2019; Piguet et al., 2021b). In this review, we mainly focus on the effect of delivery routes of rAAVs to the brain in mice and rats.

## AAV delivery routes

### Intravenous administration

AAVs are the leading vehicles for delivering gene therapies into the brain (Choudhury et al., 2017), and IV injection is the optimal brain-targeting rAAV injection route because it is minimally invasive, especially when widespread gene therapy is needed in the brain and in some disease conditions in which widely targeting both the CNS and peripheral system is required (Gessler et al., 2019). However, most rAAVs cannot cross the blood–brain barrier (BBB), and different rAAV stereotypes recognize different cell receptors and thus have different tropisms for distinct tissues and cell types (Agbandje-McKenna and Kleinschmidt, 2011; Wang et al., 2019), which causes difficulties in delivering rAAVs into the brain by IV administration. Moreover, the required high concentration of virus vectors, rapid immune responses, immunotoxicity, and potential off-targeting to the peripheral tissues may limit the use of IV for rAAV delivery into the brain (Stephenson, 2001; Gessler et al., 2019).

### rAAV9

AAV9, a naturally discovered AAV from human livers, can cross the BBB, and this makes it a useful vehicle for targeting the brain *via* IV injection (Gao et al., 2004; Foust et al., 2009) even though it is not CNS specific and can also robustly transduce peripheral tissues (Zincarelli et al., 2008; Bevan et al., 2011; Gray et al., 2011). Previous studies have shown that rAAV9 can robustly transduce neurons in rodents (Cearley and Wolfe, 2006, 2007; Cearley et al., 2008), and IV injection of self-complementary rAAV9 (scAAV9; McCarty et al., 2003) can transduce widespread neurons in the brains of neonatal mice that do not yet have a fully developed BBB (Foust et al., 2009, 2010). Moreover, IV injection of scAAV9 can transduce twice as many neurons as astrocytes, and it can transduce a small number of oligodendrocytes but not microglia in the adult mouse brain (Gray et al., 2011). Another study came to the opposite conclusion and found that IV injection of scAAV9 strongly transduced astrocytes in adult mice; however, directly injecting scAAV9 into the adult brain parenchyma caused significant transduction of neurons but not astrocytes, indicating that the injection route is critical for targeting different cells (Foust et al., 2009). Inconsistent results from previous studies may be attributed to various impurities in the viruses and different doses used in each study; thus, targeting cells *via* IV injection with rAAV9 is still controversial, and further investigation is required to validate these results.

The transduction rate after IV administration of rAAV9 may be influenced by BBB permeability (Boutin et al., 2010; Gray et al., 2011). For example, mannitol can transiently open tight junctions in the BBB. Still, it only has mild or no effect on the CNS transduction rate depending on the time points of injection of rAAV9 following injection of mannitol, which is possibly due to the active transport of rAAV9 across the BBB instead of passively passing through tight junctions (Gray et al., 2011). Consistent with this, another study showed significantly increased numbers of transduced cells and increased enzyme activity in the brain after pretreatment with mannitol (Fu et al., 2011). Moreover, pre-existing antibodies to rAAV9 capsid may limit the use of IV injection of rAAV9. Notably, preexisting immunity to rAAV9 is seen in 33.5% of the human population, although it is less common than preexisting immunity to rAAV2 (Chirmule et al., 1999; Halbert et al., 2006; Calcedo et al., 2009).

### rAAVrh.10 and rAAVrh.8

In addition to rAAV9, IV injection of rAAVrh.10, rAAVrh.39, rAAVrh.43, and rAAV7 can also lead to widespread transduction in the brain, especially rAAVth.10, which has comparable transduction ability as rAAV9 in the neonatal mouse brain (Zhang et al., 2011). Another study showed that rAAV9, rAAVrh.10, and rAAVrh.8 are superior to rAAVrh.39, rAAVrh.43, rAAV7, and rAAV8 in transduction efficiency, and rAAVrh.8 is the leading vehicle amount these vehicles in adult mice. Moreover, rAAVrh.8 transduces fewer peripheral tissues than other rAAVs, indicating that it is the optimal vehicle for delivery by IV injection to specifically transduce cells in the brain. Furthermore, these vectors transduce various cell types in the brain, such as neurons, astrocytes, and blood vessels, but not microglia, across multiple brain regions, including the cortex, striatum, hippocampus, thalamus, hypothalamus, amygdala, corpus callosum, and choroid plexus (Yang et al., 2014).

IV injection of various rAAVs has been tested in several diseases. For example, IV injection of rAAV9-*human aspartoacylase* (*hAspA*), rAAVrh.8-*hAspA*, and rAAVrh.10-*hAspA* in a neonatal mouse model of Canavan’s disease achieved widespread expression of hAspA in the brain, which alleviated neuropathy and increased the survival rate of these mice (Ahmed et al., 2013). Moreover, rAAV9 has successfully been used in various adult mouse models of lysosomal storage disorders in order to normalize enzyme expression and rescue behavior deficits (Fu et al., 2011; Ruzo et al., 2012).

### rAAV-PHP.B and rAAV-PHP.eB

AAV-PHP.B was generated by targeted evolution of Cre recombination-based AAVs, and IV injection of rAAV-PHP.B can transduce neurons, astrocytes, and oligodendrocytes, but not microglia, in widespread adult brain regions with a more than 40-fold greater transduction rate than rAAV9 (Deverman et al., 2016). Furthermore, IV injection of rAAV9 preferably transduces the liver (Pulicherla et al., 2011), while IV injection of rAAV-PHP.B transduces the CNS and liver at a similarly efficient rate. Moreover, rAAV-PHP.B transduces human neurons and astrocytes more efficiently than rAAV9. However, this effect was conditionalized by a study showing that rAAV-PHP.B’s high CNS transduction rate was seen only in the C57BL/6 J mice in which the AAV was generated, but not in BALB/cJ mice or in non-human primates, thus indicating the strain-specific property of this AAV (Deverman et al., 2016; Hordeaux et al., 2018; Matsuzaki et al., 2018). Later on, rAAV-PHP.eB, an enhanced rAAV-PHP.B variant, was developed with a lower virus dose requirement to transduce widespread cortical and striatal neurons after IV injection in C57BL/6 J mice (Chan et al., 2017). Moreover, rAAV-PHP.eB can transduce throughout the rat brain in a dose-dependent manner after IV injection (Dayton et al., 2018). However, similar to rAAV-PHP.B, rAAV-PHP.eB failed to show higher efficiency in B6C3 mice after IV injection (Mathiesen et al., 2020), thus indicating the strain specificity of rAAV-PHP.eB.

### rAAV-B1

rAAV-B1 was selected *in vivo* and shows more efficient widespread transduction efficiency than rAAV9 in the brain, including the cerebral cortex, hippocampus, thalamus, and striatum, and it induces a weaker immune response in human sera (Choudhury et al., 2016a). Furthermore, systematic injection of rAAV.B1-*acid alpha-glucosidase* (*Gaa*) showed a better treatment effect in the Pompe disease mouse model than rAAV9-*Gaa* (Keeler et al., 2019).

### rAAV-AS

AAV9.47 is a liver re-targeted variant of AAV9 (Pulicherla et al., 2011), and capsid-modified AAV9.47 was used to generate AAV-AS. rAAV-AS can transduce motor neurons and interneurons in the spinal cord, cortex, striatum, and hippocampus in the brain more efficiently than rAAV9. Notably, it can transduce 36% of striatum neurons after a single IV injection in the adult mouse brain, with similar findings also being confirmed in the adult cat brain (Choudhury et al., 2016b). Moreover, IV injection of rAAV-AS-microRNA (miRNA)-*Htt* can delete up to 50% of huntingtin (*Htt*) in different brain regions (Choudhury et al., 2016b).

### AAVHSCs

AAVHSCs are naturally occurring AAVs found in CD34-positive human peripheral blood stem cells, and they have strong tropism to CD34-positive human stem cells both *in vitro* and in mice with engraftment of human stem cells (Smith et al., 2014). Moreover, AAVHSCs can transduce widespread brain regions along with spinal cord and peripheral tissues after IV injection in non-human primates, which makes them a desirable delivery vector for targeting both the CNS and peripheral tissues (Ellsworth et al., 2019).

### Anc80L65

Anc80L65 is a novel AAV variant generated by *in silico* reconstruction, and it was first used for local delivery to the liver, muscles, and retina (Zinn et al., 2015). Later Anc80L65 was demonstrated to efficiently transduce neurons and astrocytes in the brain and spinal cord after IV injection in mice (Hudry et al., 2018).

### Newly discovered or engineered AAVs in the past 3 years

More promising AAV9 variants have been discovered or engineered in recent years. For example, rAAV-F shows 65-fold and 171-fold higher transgene expression in astrocytes and neurons, respectively, than its parental rAAV9 after IV injection in mice (Hanlon et al., 2019). One study developed rAAV-PHP.V1 with brain vascular cell tropism and rAAV-PHP.N with specific neuron tropism (Ravindra Kumar et al., 2020). Ten AAV9 variants selected using the TRACER (Tropism Redirection of AAV by Cell-type-specific Expression of RNA) platform showed a higher brain transduction rate than AAV9 (13-fold to 385-fold) and a lower transduction rate in the peripheral tissues such as in the liver after IV injection in mice. Among these vectors, AAV-9P31 has the most striking brain transduction rate and can achieve a more than 1,000-fold higher transduction rate in the spinal cord than AAV9 after IV injection in mice (Nonnenmacher et al., 2021). Also, IV injection of the AAV9 variants MaCPNS1/2 can achieve a higher transduction rate for astrocytes and neurons in various brain regions compared with rAAV9 in non-human primates (Chen et al., 2022). It is important in future work to validate these novel AAV vectors in various disease models.

### Using CNS-specific promoters in rAAVs to increase CNS specificity after IV injection

The CNS can be specifically targeted using CNS-specific promoters; however, these promotors are usually too big to introduce into AAVs. Moreover, changing cellular states may change the promotor activities, and cells in the peripheral nervous system might share the same promotors as in the CNS (Foust et al., 2009). Furthermore, cellular or tissue-specific promoters usually express limited genes and fail to achieve therapeutic effects, thus robust ubiquitous promotors, such as CMV-enhancer/chicken β-actin promoters, are commonly used in vector design.

### Introducing binding sites of peripheral miRNAs increases CNS specificity following IV injection of rAAVs

Another way to specifically target the brain after IV injection is to use endogenous miRNAs. miRNAs can reduce the stability of mRNA or cleave the mRNA by partially or completely binding to the mRNA, respectively (Bartel, 2009), and thus introducing binding sites for miRNAs in the rAAVs can suppress the expression of rAAV-associated genes in specific tissues expressing these miRNAs. The liver, heart, and skeletal muscle are the most commonly off-targeted tissues following IV injection of rAAV9 (Pacak et al., 2006; Zincarelli et al., 2008). Therefore, introducing the binding site of miR122, which is expressed in hepatocytes (Lagos-Quintana et al., 2002), and/or the binding site of miR-1, which is expressed in heart and skeletal muscles (Chang et al., 2004), can suppress vector expression in these tissues and thus target the CNS more specifically (Xie et al., 2011). Another study successfully used rAAV9-*hAspA*-*miRBS* with an miRNA-regulated vector genome to silence the peripheral expression of *hAspA* (Ahmed et al., 2013). Moreover, IV injection of rAAVrh.10-*Egfp*-*miRBS* showed efficient CNS transduction and limited peripheral transduction in adult marmosets (Yang et al., 2014). Furthermore, inserting an miR183 biding site in rAAVs can prevent dorsal root ganglion toxicity (Hordeaux et al., 2020). Therefore, introducing different peripheral miRNA binding sites is a powerful tool for more specifically targeting the CNS and minimizing the peripheral off-target side effects.

## Stereotaxic intraparenchymal injection

**Figure 1:**
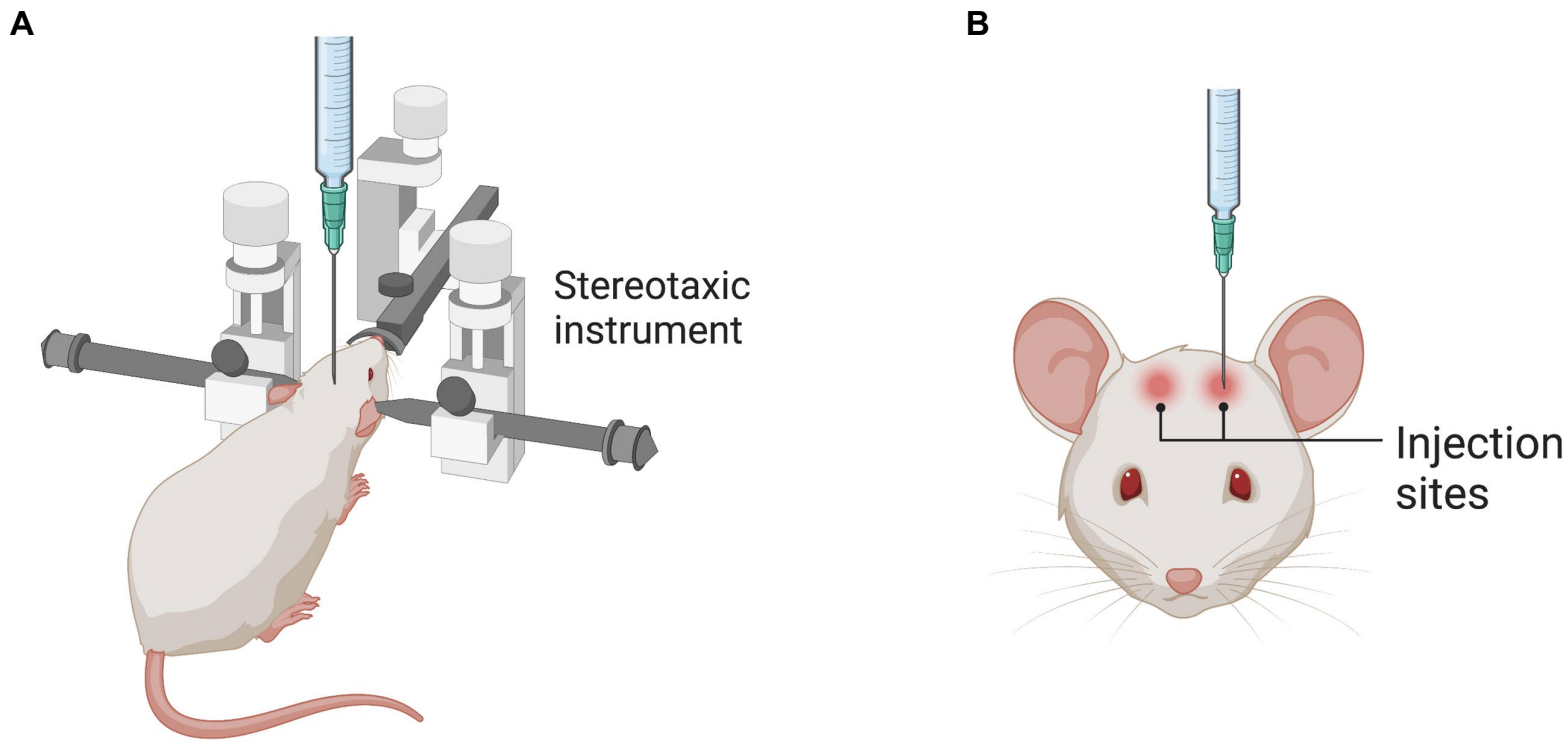
Stereotaxic injection into the rodent brain. **(A)** The illustration shows the stereotaxic instrument used to locate the specific brain regions. **(B)** The illustration shows the injection sites in the brain as an example.

Stereotaxic intraparenchymal injection using stereotaxic coordinates (Figure 1) is the most widely used brain gene therapy delivery strategy. Micropipettes and automated pumps are used to precisely deliver the dose and minimize injection-induced injury. Notably, intraparenchymal injection requires smaller numbers of rAAVs and causes less immune response than IV and cerebrospinal fluid (CSF) deliveries (McPhee et al., 2006; Zerah et al., 2015). However, this technique may cause brain injury; for example, intraluminal injection increased the risk of intracranial hemorrhage and self-limited headache in a clinical trial (Christine et al., 2009).

rAAVs start to diffuse and transduce targeted cells after injection into the targeted brain regions. However, AAVs only diffuse a relatively short distance and can only be expressed in limited areas around the injection sites, and thus multiple injection locations are required to reach the desired coverage of the therapeutic areas (Vite et al., 2005; Worgall et al., 2008; Leone et al., 2012). Some gene therapy-expressed therapeutic proteins – such as lysosome enzymes – can spread *via* axonal transport (Baek et al., 2010). Moreover, rAAV vectors can be transported by anterograde and retrograde axonal transport (Boulis et al., 2003; Kaspar et al., 2003), which is serotype dependent (Salegio et al., 2013; Green et al., 2016; Table 1). For example, rAAV9 can be transported in neurons retrogradely and anterogradely driven by cytoplasmic dynein and kinesin 2, respectively. Moreover, increasing the amount of rAAV9 can increase rAAV9 transport, indicating that an increased dose of rAAV9 may improve its distal distribution (Castle et al., 2014). Furthermore, engineered AAV2-Retro and AAV9-Retro have greater retrograde transport capacity than AAV9, and this provides new tools for efficiently targeting neural circuits (Tervo et al., 2016; Lin et al., 2020). Convection-enhanced delivery (CED) is a technology using pressure gradients to increase the diffused area of the delivered molecules or rAAVs. It can enhance delivery of small and large molecules from millimeters to centimeters (Bobo et al., 1994; Morrison et al., 1994), and it can be used to increase the distribution of the rAAVs and is especially useful for large animals (Bankiewicz et al., 2000; Hadaczek et al., 2006; Mehta et al., 2017).

**Table 1:** Anterograde and retrograde axonal transport of various rAAVs in different brain regions.

Serotypes	Injected position	Anterograde and retrograde axonal transport to	References
rAAV2	Hippocampus	Distal brain regions	Kaspar et al. (2002), Passini et al. (2002), Hennig et al. (2003)
rAAV2	Hippocampus	Entorhinal cortex	Kaspar et al. (2002), Passini et al. (2005)
rAAV2	Hippocampus	Medial septum and contralateral hippocampus	Passini et al. (2005)
rAAV9	Hippocampus	Contralateral hippocampus, the septal nuclei, and the entorhinal cortex	Cearley and Wolfe (2006, 2007)
rAAV2	Striatum	Substantia nigra	Kaspar et al. (2002), Cearley and Wolfe (2007)
rAAV9	Striatum	Amygdala, thalamus, and substantia nigra	Cearley and Wolfe (2007)
rAAVrh.10	Striatum	Frontal cortex, thalamus, and substantia nigra	Sondhi et al. (2007), Cearley and Wolfe (2007)
rAAVrh.10	Striatum	Hippocampus	Winner et al. (2016), Gray et al. (2019)
rAAV-TT	Striatum	Corpus callosum, cortex, thalamus, and substantia nigra	Tordo et al. (2018)
rAAV1 or rAAV2	Deep cerebellar nuclei	Cerebellar cortex, hindbrain, brain stem, and spinal cord	Dodge et al. (2008)
rAAV9	Ventral tegmental area	Striatum, habenula, retrosplenial cortex, thalamus, and endopiriform nuclei	Cearley and Wolfe (2007)

All cell types in the brain parenchyma are potential targets for gene therapy in different diseases. However, sometimes only one cell type is intended to be targeted in one specific disease, for example, neurons in Huntington’s disease (Yang et al., 2017) and astrocytes in astrocyte-to-neuron conversion studies of neurodegenerative diseases (Wang et al., 2021). Therefore, cell-specific targeting is critical to achieving the intended therapeutic effects and minimizing off-target responses. One strategy to specific target one cell type, as we discussed above, is to use cell-specific promoters for example, using glial fibrillary acidic protein (GFAP) promoter to increase astrocyte specificity (Chen et al., 2020). Another strategy is to discover or develop AAVs with specific cell tropism. For example, the engineered AAV variants ShH13, ShH19, and L1-12 showed a higher astrocytes transduction rate than AAV2 and AAV6; however, AAV9 was not compared in this study (Koerber et al., 2009). Moreover, the AAV variant Olig001 was developed that has strong oligodendrocyte tropism (Powell et al., 2016; Mandel et al., 2017). Furthermore, a recent study found that an AAV9 variant, AAV-cMG, could efficiently and safely transduce microglia (Lin et al., 2022). Therefore, different promoters and AAV serotypes can be combined to specifically target one specific cell type in the brain parenchyma.

### Intrahippocampal viral injection

rAAV2 can transduce a large number of neurons in the hippocampus, especially in the dentate hilus, but with a very limited transduction rate in the dentate granule neurons (Bartlett et al., 1998; Kaspar et al., 2002). Apart from local diffusion, intrahippocampal rAAV2 can be transported to other brain regions through anterograde and retrograde transport. For example, hippocampal hilus mossy cells project into the hippocampal molecular layer of both hemispheres, and green fluorescent protein (GFP) can be anterogradely transported to both ipsilateral and contralateral projections of hippocampal hilus mossy cells 2 weeks after intrahippocampal rAAV2-*Gfp* injection (Kaspar et al., 2002), and anterograde transport of lysosome enzymes leads to their presence in distal brain regions (Passini et al., 2002; Hennig et al., 2003). Some studies have shown that retrograde transport of rAAV2 takes place when more than 10^10^ genome vectors are injected (Kaspar et al., 2003; Passini et al., 2005), while another study showed low retrograde transport using large amounts of rAAV2 (Burger et al., 2004). The method of rAAV preparation might be a critical factor affecting retrograde transport, and pathological neurons may have reduced ability for retrograde transport (Walkley, 1998; Passini et al., 2005).

Notably, rAAV2 vectors were shown to be robustly detected in the entorhinal cortex layer II neurons that project to the hippocampus, probably due to retrograde transport after intrahippocampal injection (Kaspar et al., 2002). Entorhinal cortex layer II neurons are largely degenerated during the early development of Alzheimer’s disease, and retrograde targeting of these neurons by intrahippocampal injection of rAAV2-*Bcl2l* can prevent the death of entorhinal cortex layer II neurons, which may slow down the disease progression. Moreover, entorhinal cortex layer II neurons are widely anatomically distributed and thus are difficult to target by direct injections, and retrograde targeting provides an attractive method to target all of these neurons after intrahippocampal injection. Unilateral hippocampal injection of rAAV2-*human acid sphingomyelinase* (*hASM*) results in protein and mRNA expression of *hASM* in the ipsilateral and contralateral hippocampus, entorhinal cortex, and medial septum, and this has been used to correct Niemann-Pick disease (a fatal lysosomal storage disease; Passini et al., 2005).

rAAV9 can retrogradely transduce neurons after intrahippocampal injection; for example, intrahippocampal injection of Cy3-labeled rAAV9 can transduce not only various regions of the hippocampus, including the dentate gyrus, pyramidal cell layer, and oriens cell layer, but can also transduce cells in the entorhinal and retrosplenial cortex, possibly due to retrograde transport of the vectors (Cearley and Wolfe, 2007). Moreover, both the β-glucuronidase (GUSB) enzyme and mRNA can be detected in the contralateral hippocampus, the septal nuclei, and the ipsilateral entorhinal cortex after injection of rAAV9-*Gusb*, indicating the possible retrograde transport of the vectors, and only the enzyme, and not the mRNA, was found in the contralateral entorhinal cortex, indicating the presence of axonal enzyme transport (Cearley and Wolfe, 2006). Notably, CED can enhance the volume of distribution of various AAV vectors, and rAAV9 outperforms rAAV5 and rAAV 8 after intrahippocampal injection by CED in the adult mouse brain (Carty et al., 2010).

Furthermore, vector expression was detected in the CA1 region of both the ipsilateral and contralateral hemispheres after CA2/3 injection of rAAV9, and the expression in contralateral CA1 may be attributed to anterograde transport because CA3 sends projections to CA1 in both hemispheres. The vector expression was also detected in septal nuclei, which might be due to the retrograde transport and anterograde transport to medial septal nuclei and lateral septal nuclei, respectively, because the medial septal nuclei send projections to CA2/3 and the lateral septal nuclei receive projections from ipsilateral CA3. However, no vector expression was seen in the entorhinal cortex due to its weak projection to CA2/3 (Cearley and Wolfe, 2007). In the same study, after injection of rAAV9 into the dentate gyrus vector expression was detected in the entorhinal cortex and medial septal nuclei, which is possibly due to retrograde and anterograde transport, respectively, because of the large number of projections from the entorhinal cortex to the dentate gyrus and projections from the dentate gyrus to the medial septal nuclei (Steward, 1976; Wyss, 1981).

### Intrastriatal viral injection

rAAV2, rAAV9, and rAAVrh.10 can strongly transduce striatum neurons after intratriatumic injection (Fan et al., 1998; Mandel et al., 1998; Lo et al., 1999; Cearley and Wolfe, 2007; Boussicault et al., 2016), and they can be retrogradely transported to and then transduce the neurons in the substantia nigra pars compacta that project to the striatum (Kaspar et al., 2002; Cearley and Wolfe, 2007). The degeneration of dopaminergic nigrostriatal neurons causes the clinical manifestations of Parkinson’s disease (Lew, 2007), and thus intrastriatal injection of rAAVs can provide a new injection route for targeting the substantia nigra pars compacta. The advantage of using retrograde targeting of the substantia nigra for Parkinson’s disease is that it can deliver therapeutic genes to both the striatum and substantia nigra after one injection because the substantia nigra is not easily located by direct injection. Moreover, rAAV9 can also be retrogradely transported to the amygdala and thalamus, which have projections to the striatum (Cearley and Wolfe, 2007).

One study showed that rAAV5 can transduce both neurons and astrocytes after intrastriatal injection and that rAAV2 can transduce only neurons, while rAAV4 does not transduce any parenchymal cells after intrastriatal injection (Davidson et al., 2000). Heparan sulfate proteoglycan (HSPG) has been reported to be a primary receptor for rAAV2 binding (Summerford and Samulski, 1998) and extracellular HSPG binds with rAAV2, thus limiting the distribution and conduction efficiency of rAAV2, which might explain why rAAV5 shows wider distribution after injection than rAAV2 (Davidson et al., 2000). But the volume of distribution of rAAV2 can be enhanced by CED in the rat brain (Nguyen et al., 2001). Moreover, intrastriatal injection of rAAV5 carrying miRNA targeting *Htt* (AAV5-*miHtt*) transcripts can achieve widespread miRNA-*Htt* in the striatum and parts of the cortex of rats, mice, and large animal models, thus preventing mutant *Htt* aggravation and neuronal dysfunction (Miniarikova et al., 2016, 2017; Evers et al., 2018).

rAAVrh.10 outperforms rAAV2, rAAV5, and rAAV8 in terms of brain distribution after intrastriatal injection of these vectors carrying *tripeptidyl peptidase I* (*Tpp-I*), and the projecting neurons in the frontal cortex, thalamus, and substantia nigra also express rAAVrh.10-*Tpp-I* after the injection, indicating the retrograde transport of rAAVrh.10-*Tpp-I*. Moreover, a single rAAVrh.10-*Tpp*-1 injection reduces the lysosome storage granules and improves behavior recovery in the mouse model of infantile neuronal ceroid lipofuscinosis (a lysosome disorder; Sondhi et al., 2007). Moreover, intrastriatal injection of rAAVrh.10-*sulfoglucosamine sulfohydrolase* (*Sgsh*) transduces neurons in the striatum and hippocampus, restores *Sgsh* expression in these regions, and ameliorates both GM3 accumulation in the injection site and the disease pathology in the adult mucopolysaccharidosis (MPS) IIIA mouse model (Winner et al., 2016; Gray et al., 2019).

A new variant called AAV-TT was generated based on AAV2. Intrastriatal injection of rAAV-TT outperforms AAV9 and rAAVrh.10 and transduces widespread brain regions, including the corpus callosum, cortex, thalamus, and substantia nigra pars compacta in adult mice (Tordo et al., 2018). Moreover, intrastriatal injection of a low dose of rAAV-TT-*heparan sulphate acetyl-CoA:α-glucosaminide N-acetyltransferase* (*Hgsnat*), but not rAAV9-*Hgsnat*, can correct MPS III mouse pathological phenotypes (Tordo et al., 2018).

### Deep cerebellar nuclei injection

DCN receive neuronal projections from all parts of the spinal cord (Matsushita and Yaginuma, 1995; Matsushita and Gao, 1997; Matsushita, 1999a,b). Bilateral injection of rAAV1 or rAAV2-*insulin-like growth factor 1* (*Igf-1*) into DCN results in IGF expression throughout the cerebellar cortex, hindbrain, brain stem, and spinal cord, and it delays the progression of the amyotrophic lateral sclerosis (ALS) mouse model, providing a novel gene therapy delivery route for ALS (Dodge et al., 2008). rAAV1 outperforms rAAV 2, 5, 7, and 8 in expressing the encoding genes after unilateral DCN injection, and the expression of *hASM* from rAAV1-*hASM* can be detected throughout the cerebellum, brain stem, midbrain, and spinal cord. Moreover, bilateral injection of rAAV1-*hASM* alleviates storage of lysosomal sphingomyelin and further corrects the behavioral deficits (Dodge et al., 2005).

### Ventral tegmental area injection

The VTA comprises a group of neurons projecting into various brain regions and receives divergent efferent neurons from different parts of the brain. VTA injections of rAAV1, rAAV9, and rAAVrh.10 achieve wide spreads of virus vectors across the brain, with rAAV9 being the most widely distributed vector. rAAV9 can be retrogradely transported after VTA injection to all areas with projections to the VTA, including the striatum, habenula, retrosplenial cortex, thalamus, and endopiriform nuclei, and a single injection of rAAV9-*Gusb* can correct the lysosomal disorders in all brain regions in the MPS VII mouse model (Cearley and Wolfe, 2007). Notably, the authors further showed that intrahippocampal and intrastriatal injections have much more limited rAAV9 transduction areas than the VTA.

## Delivery into the CSF

### Intracerebroventricular injection

*ICV injections* of rAAV2, rAAV4, and rAAV5 transduce primary ependymal cells in the choroid plexus but not in other parts of the brain in adult mice and rats (Bajocchi et al., 1993; McCown et al., 1996; Lo et al., 1999; Davidson et al., 2000). However, other studies have shown that rAAV2 transduction can be observed in the hypothalamus after ICV injection in adult rats, although the transduction rate is limited (Alexander et al., 1996; Rosenfeld et al., 1997; Wu et al., 1998). Furthermore, ICV injection of rAAV9-iduronate-2-sulfatase decreases brain lesions and shows long-term cognitive improvement in the MPS II adult mouse model (Hinderer et al., 2016).

Intraventricular injections of rAAV2 in neonatal mice can achieve a global distribution in the brain parenchyma, especially in principle neurons such as granule cells in the hippocampus, mitral cells in the olfactory bulb, and Purkinje cells in the cerebellum, *via* its circulation in the subarachnoid space in the CSF (Passini and Wolfe, 2001). rAAV1 also shows widespread transduction in neonatal mice after lateral ventricular injection with different target regions compared to rAAV2. For example, rAAV1 transduces more cells in the neocortex, entorhinal cortex, and CA1 and CA3 of the hippocampus, but fewer cells in the dentate gyrus of the hippocampus, thalamus, and superior colliculus, which makes rAAV2 and rAAV1 complementary. Moreover, rAAV1-glucuronidase reverses the pathology in neonatal mice with MPS VII (Passini et al., 2003; Piguet et al., 2021a), while rAAV1-βgal reverses the pathology of GM1-gangliosidosis after ICV injection in a neonatal mouse model of the disease (Broekman et al., 2007).

rAAV8 shows more efficient transduction than rAAV1 and rAAV2 and can significantly transduce the cerebral cortex, hippocampus, cerebellum, and olfactory bulb after ICV injection in neonatal mice (Broekman et al., 2006). Furthermore, the human α-L-iduronidase gene (*Idua*) can be detected in multiple brain regions, including the hippocampus, corpus callosum, cortex, caudate-putamen, and cerebellar Purkinje cell layer after ICV injection of rAAV8-*Idua* in a neonatal MPS I mouse model, and this reduces the accumulation of glycosaminoglycan and prevents cognitive dysfunction (Wolf et al., 2011). Furthermore, ICV injection of a low dose of rAAV9-, rAAVrh.8-, and rAAVrh.10-*Aspa* can increase the overall survival rate in a neonatal mouse model of Canavan’s disease but does not rescue the motor deficits, which is possibly due to a role for peripheral tissues in disease pathology (Ahmed et al., 2016).

SCH9, a novel AAV variant, can efficiently transduce neural stem cells with 24-fold greater GFP expression and 12-fold greater transduction volume compared with AAV9 after ICV injection in mice (Ojala et al., 2018). Another novel AAV variant, Anc80L65, spread throughout the brain more broadly than AAV9 and could even efficiently transduce cells in the cerebellum after ICV injection in mice (Hudry et al., 2018).

### Intracisternal and intralumbar injection

Intracisternal injection of rAAV9-*Sgsh* restores *Sgsh* expression in widespread brain regions and somatic tissues in adult MPS IIIA mice, which prevents the pathology in both the CNS and peripheral tissues and normalizes the behavioral deficits in these mice (Haurigot et al., 2013). Moreover, GFP can be detected in cerebellar Purkinje cells, the medulla, and discrete nuclei after intracisternal and intralumbar injection of rAAV9-GFP in 5-day-old pigs (Bevan et al., 2011). Studies showed that rAAV-PHP.eB was superior to rAAV9 in brain transduction rate after intracisternal injection in adult rats and after intrathecal injections in non-human primates (Arotcarena et al., 2021; Chatterjee et al., 2022). However, the PHP.eB variant raises concerns about their species and strain specificity as discussed above. Notably, intralumbar injection is usually used in large animals and is less efficient at brain transduction than intracisternal and intraventricular injections (Hinderer et al., 2018; Piguet et al., 2021a).

The Trendelenburg position after intracisternal or intralumbar injection has been shown to enhance virus transduction to the brain and spinal cord (Borel et al., 2018; Cain et al., 2019). However, one study showed that transduction efficiency was not improved using a 10-min Trendelenburg position after intralumbar injection in non-human primates (Hinderer et al., 2018). Despite these contrasting results, a 2-h Trendelenburg position following intracisternal injections was shown to achieve a 15-fold increase in transduced neurons, increased transduction in cortical regions, and increased consistency of gene expression compared to an upright position in adult rats (Castle et al., 2018). Moreover, a 6-min Trendelenburg position after intralumbar injection of rAAV9 achieved widespread spinal cord and brain transduction in adult mice (Bey et al., 2017). Taken together, the Trendelenburg position appears to be an effective method to enhance brain and spinal cord AAV transducing rate and distribution. However, different durations of the Trendelenburg position should be tested in different species and under different experimental settings.

## Intranasal administration

Transduced cells are limited to the nasal epithelium and olfactory bulb after intranasal administration of rAAV9-*Idua* in an adult MPS I mouse model; however, accumulated pathogenic materials are reduced in the brain due to enzyme diffusion from transduced cells into the brain (Belur et al., 2017). Similar results have also been obtained in *Idua*-deficient mice after nasal administration of rAAV9-*Idua* (Wolf et al., 2012). Intranasal administration of rAAV2-*brain-derived neurotrophic factor* (*Bndf*) enhances the level of BNDF in the hippocampus and prevents depression in these mice (Ma et al., 2016).

## Conclusion

Different administration routes of rAAVs may affect the transduction rate and even the cellular types that are transduced in the brain. Therefore, different administration routes of rAAVs need to be carefully selected for different CNS diseases. For example, in some disorders AAV transduction in broad brain regions is required, such as in lysosomal disorders, and thus administration routes that can achieve widespread brain transduction are preferred, such as IV injection, delivery into the CSF, and intraparenchymal injections with retrograde and anterograde transport of the virus or enzymes. However, in some other diseases specific neuronal regions need to be targeted, such as in Parkinson’s disease and Huntington’s disease, and in these cases intraparenchymal injection may be the optimal administration route.

Each administration route has its benefits and shortcomings. For example, IV injection is less invasive and can be used to target widespread areas of the brain; however, the currently used rAAV serotypes lack robust brain tropism and may induce immunotoxicity. Notably, inserting a peripheral miRNA binding site can be a solution to increasing the brain specificity. Nonetheless, new serotypes or engineered rAAVs with robust brain tropism, less immunogenicity, and less immunotoxicity need to be discovered or generated. Moreover, a large dose of rAAVs is usually required for IV injection, and thus the manufacturing capacity and costs can be challenging. Intraparenchymal injection can achieve efficient neuron transduction in the injection site and in distal regions by retrograde and anterograde transport; however, no AAVs have been shown to target microglia for microglia-induced or microglia-involved diseases such as hereditary diffuse leukoencephalopathy with spheroids and most, if not all, neurodegenerative diseases. rAAV delivered to the CSF can widely transduce the brain in neonatal mice and rats, while the transduction rate is low in adult animals. Moreover, intraparenchymal, intracisternal, and ICV injection may cause CNS injury and hemorrhage in the brain.

The efficiency and safety of distinct administration routes of rAAVs need to be tested in large animals after the proof of concept in rodents. The human brain is 1,000 times larger than the mouse brain and has different anatomy, and the distance between brain regions to the ventricle for ICV injection is much greater in large animals and humans compared to rodents. Therefore, it is essential to conduct studies using large animals to design optimal clinical trial protocols.

## Author contributions

KZ wrote the manuscript draft and prepared the figures. All other authors edited and revised the manuscript, and read and approved the final manuscript for publication.

## Funding

This study was supported by the National Nature Science Foundation of China (U21A20347), the Swedish Childhood Cancer Foundation (PR2018-0082, PR2021-0020), the Swedish Cancer Foundation (20-1121-PjF), and Swedish Governmental grants to scientists working in health care (ALFGBG-965197).

## Conflict of interest

The authors declare that the research was conducted in the absence of any commercial or financial relationships that could be construed as a potential conflict of interest.

## Publisher’s note

All claims expressed in this article are solely those of the authors and do not necessarily represent those of their affiliated organizations, or those of the publisher, the editors and the reviewers. Any product that may be evaluated in this article, or claim that may be made by its manufacturer, is not guaranteed or endorsed by the publisher.

## Glossary

CNS: Central nervous system
AAV: Adeno-associated virus
rAAV: Recombinant Adeno-associated virus
AAVrh.10: AAV rhesus isolate 10
IV: Intravenous
BBB: Blood–brain barrier
scAAV9: Self-complementary rAAV9
hAspA: Human aspartoacylase
Gaa: Acid alpha-glucosidase
miRNA: MicroRNAs
hASM: Human acid sphingomyelinase
Gusb: β-glucuronidase gene
Htt: Huntingtin
Tpp-I: Tripeptidyl peptidase I
Sgsh: Sulfoglucosamine sulfohydrolase
MPS: Mucopolysaccharidosis
Hgsnat: Heparan sulphate acetyl-CoA:α-glucosaminide N-acetyltransferase
DCN: Deep cerebellar nuclei
Igf-1: Insulin-like growth factor 1
ALS: Amyotrophic lateral sclerosis
VTA: Ventral tegmental area
CSF: Cerebrospinal fluid
CED: Convection-enhanced delivery
ICV: Intracerebroventricular
Idua: α-L-iduronidase gene
Bndf: Brain-derived neurotrophic factor
